# Renin-Angiotensin-Aldosterone System Inhibitors and Development of Gynecologic Cancers: A 23 Million Individual Population-Based Study

**DOI:** 10.3390/ijms24043814

**Published:** 2023-02-14

**Authors:** Nhi Thi Hong Nguyen, Phung-Anh Nguyen, Chih-Wei Huang, Ching-Huan Wang, Ming-Chin Lin, Min-Huei Hsu, Hoang Bui Bao, Shuo-Chen Chien, Hsuan-Chia Yang

**Affiliations:** 1 School of Health Care Administration, College of Management, Taipei Medical University, Taipei 11031, Taiwan; 2 Health Personnel Training Institute, University of Medicine and Pharmacy, Hue University, Hue 491-20, Vietnam; 3 Clinical Data Center, Office of Data Science, Taipei Medical University, Taipei 106339, Taiwan; 4 Clinical Big Data Research Center, Taipei Medical University Hospital, Taipei 11031, Taiwan; 5 International Center for Health Information Technology (ICHIT), College of Medical Science and Technology, Taipei Medical University, Taipei 106339, Taiwan; 6 Graduate Institute of Biomedical Informatics, College of Medical Science and Technology, Taipei Medical University, Taipei 106339, Taiwan; 7 Biomedical Informatics & Data Science (BIDS) Section, School of Medicine, Johns Hopkins University, 2024 E Monument St, Suite 1-200, Baltimore, MD 21205, USA; 8 Department of Neurosurgery, Shuang Ho Hospital, Taipei Medical University, New Taipei City 235041, Taiwan; 9 Taipei Neuroscience Institute, Taipei Medical University, Taipei 11031, Taiwan; 10 Graduate Institute of Data Science, College of Management, Taipei Medical University, Taipei 11031, Taiwan; 11 Internal Medicine Department, University of Medicine and Pharmacy, Hue University, Hue 491-20, Vietnam; 12 Research Center of Big Data and Meta-Analysis, Wan Fang Hospital, Taipei Medical University, Taipei 116079, Taiwan

**Keywords:** renin-angiotensin-aldosterone system, ACEIs, ARBs, gynecologic cancer risk, cervical cancer, endometrial cancer, ovarian cancer

## Abstract

The chronic receipt of renin-angiotensin-aldosterone system (RAAS) inhibitors including angiotensin-converting enzyme inhibitors (ACEIs) and angiotensin receptor blockers (ARBs) have been assumed to be associated with a significant decrease in overall gynecologic cancer risks. This study aimed to investigate the associations of long-term RAAS inhibitors use with gynecologic cancer risks. A large population-based case-control study was conducted from claim databases of Taiwan’s Health and Welfare Data Science Center (2000–2016) and linked with Taiwan Cancer Registry (1979–2016). Each eligible case was matched with four controls using propensity matching score method for age, sex, month, and year of diagnosis. We applied conditional logistic regression with 95% confidence intervals to identify the associations of RAAS inhibitors use with gynecologic cancer risks. The statistical significance threshold was *p* < 0.05. A total of 97,736 gynecologic cancer cases were identified and matched with 390,944 controls. The adjusted odds ratio for RAAS inhibitors use and overall gynecologic cancer was 0.87 (95% CI: 0.85–0.89). Cervical cancer risk was found to be significantly decreased in the groups aged 20–39 years (aOR: 0.70, 95% CI: 0.58–0.85), 40–64 years (aOR: 0.77, 95% CI: 0.74–0.81), ≥65 years (aOR: 0.87, 95% CI: 0.83–0.91), and overall (aOR: 0.81, 95% CI: 0.79–0.84). Ovarian cancer risk was significantly lower in the groups aged 40–64 years (aOR: 0.76, 95% CI: 0.69–0.82), ≥65 years (aOR: 0.83, 95% CI: 0.75–092), and overall (aOR: 0.79, 95% CI: 0.74–0.84). However, a significantly increased endometrial cancer risk was observed in users aged 20–39 years (aOR: 2.54, 95% CI: 1.79–3.61), 40–64 years (aOR: 1.08, 95% CI: 1.02–1.14), and overall (aOR: 1.06, 95% CI: 1.01–1.11). There were significantly reduced risks of gynecologic cancers with ACEIs users in the groups aged 40–64 years (aOR: 0.88, 95% CI: 0.84–0.91), ≥65 years (aOR: 0.87, 95% CI: 0.83–0.90), and overall (aOR: 0.88, 95% CI: 0.85–0.80), and ARBs users aged 40-64 years (aOR: 0.91, 95% CI: 0.86–0.95). Our case-control study demonstrated that RAAS inhibitors use was associated with a significant decrease in overall gynecologic cancer risks. RAAS inhibitors exposure had lower associations with cervical and ovarian cancer risks, and increased endometrial cancer risk. ACEIs/ARBs use was found to have a preventive effect against gynecologic cancers. Future clinical research is needed to establish causality.

## 1. Introduction

Cervical, endometrial, and ovarian carcinomas make up the majority of tumors in gynecologic cancers [1]. Cervical cancer was reported as the most common in all gynecologic cancers, with more than 604,120 new cases and 341,830 new deaths diagnosed in 2020 [2,3]. The evidences indicated that ovarian cancer accounted for the highest fatality rate among gynecological malignancies due to silent progression and advanced stage at diagnosis [4,5,6]. There were nearly 320,000 new cases and 207,000 new deaths recorded in ovarian cancer [2]. Endometrial cancer ranked sixth among female cancers, with over 417,000 new cases [7]. The most common female gynecologic malignancies in Taiwan were uterine body, ovary, and other adnexa, and cervix cancers [8]. While the incidence rate of cervix uterine cancers increased until 80 years, those uterine body and ovarian cancers reached a peak at 50 and 60 years, respectively.

The circulating renin–angiotensin-aldosterone system (RAAS) is primarily known for its pivotal role in regulating aldosterone secretion, blood pressure, cardiovascular homeostasis, fluid volume, and electrolyte balance [9,10,11]. Both angiotensin-converting-enzyme inhibitors (ACEIs) and angiotensin receptor blockers (ARBs) are commonly used and regarded as safe therapies with few side effects [10]. However, there is an increasing evidence that long-term drugs affecting the RAAS may have impacts on the risk of cancers [12], including gynecological cancers [13,14,15]. Numerous observational studies on the associations of ARBs and ACEIs with gynecological cancers have produced contradictory findings. Some studies indicated a higher overall incidence of cancer among ARB users [13], whereas others found a lowered risk of disease progression and lower recurrence in ovarian cancer [16]. In addition, previous studies demonstrated that women who used ACEIs had decreased rates of gynecologic tract cancer [15], while others highlighted that individuals with ovarian cancer had higher serum ACEI levels. Circulating ACEIs may be linked to ongoing pathobiologic processes in the development of ovarian cancer [17] and endometrial cancer [14]. Some evidence has indicated that RAAS inhibitors may affect angiogenesis, tumor cell proliferation, follicle maturation, cell proliferation, and vascularization in gynecological human tissues both in vitro and in vivo [1,18,19,20,21]. Therefore, long-term intake of RAAS inhibitors has increased apprehensions [20].

To our knowledge, a few studies have been conducted on gynecologic cancer risks in RAAS inhibitors users and stratified by age. This study aimed to investigate the associations of long-term RAAS inhibitors use with gynecological cancer risks in particular age groups.

## 2. Results

### 2.1. Descriptive Analysis

**Figure 1:**
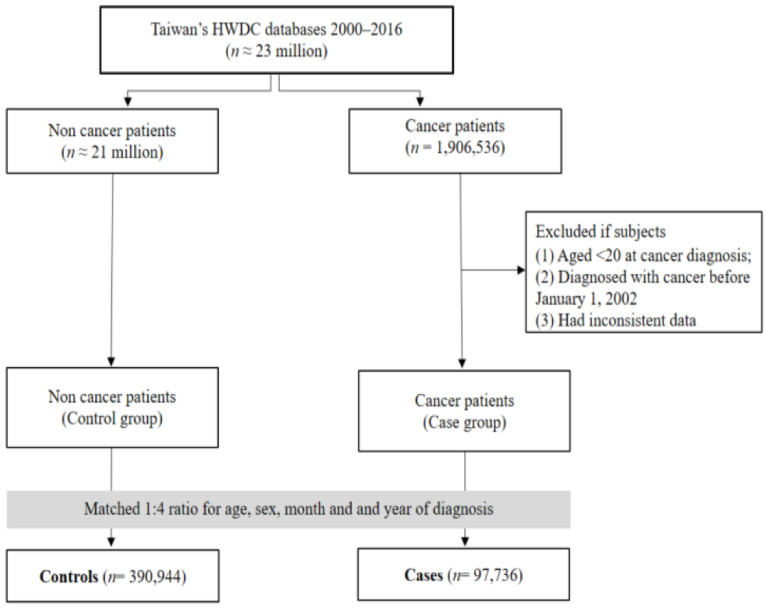
Workflow of the case-control study design.

A total of 97,736 gynecologic cancer cases, including 64,382 cases of cervical cancer, 19,580 cases of endometrial cancer, and 13,774 cases of ovarian cancer, were identified between 2002 and 2016. After each case was matched with four controls, there were 390,944 patients without any cancer diagnosis as control group. The number of control individuals with cervical, endometrial, and ovarian cancers was 257,528, 78,320, and 55,096, respectively (Figure 1). The average age of gynecologic cancer cases and controls was 50.81 years (Table 1). The individuals aged 40–64 years was dominant in gynecologic cancers, consisting of 59.41%. The case group had higher rates of diabetes (14.19%) and peptic ulcer disease (12.66%) than the control group, which were higher by 2.3% and 2.18%, respectively. The case group used metformin, aspirin, and statins more frequently than the control group by 1.58%, 1.29%, and 2.45%, respectively (Table 1).

### 2.2. Association of RAAS Use with Overall Gynecologic Cancer

**Figure 2:**
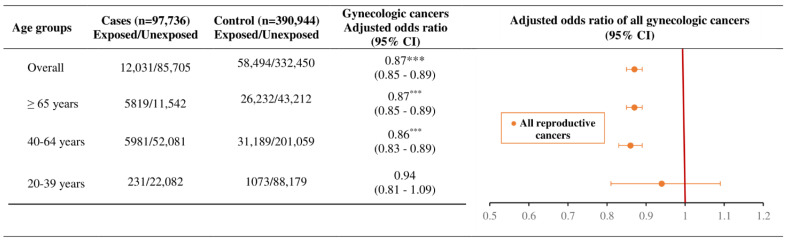
The association of RAAS inhibitors use with overall gynecologic cancer risk by age groups with adjusted odds ratio. Footnote: *** *p* < 0.0001.

Figure 2 indicates the associations of RAAS inhibitors intake and gynecologic cancers by age groups. RAAS medication use was associated with a decreased risk of gynecologic cancers (adjusted odds ratio (aOR): 0.87, 95% CI: 0.85–0.89). The degree of gynecologic cancer risk was observed to have significant associations with RAAS users aged 40–64 years (aOR: 0.86, 95% CI: 0.83–0.89) and ≥65 years (aOR: 0.87, 95% CI: 0.85–0.89).

**Figure 3:**
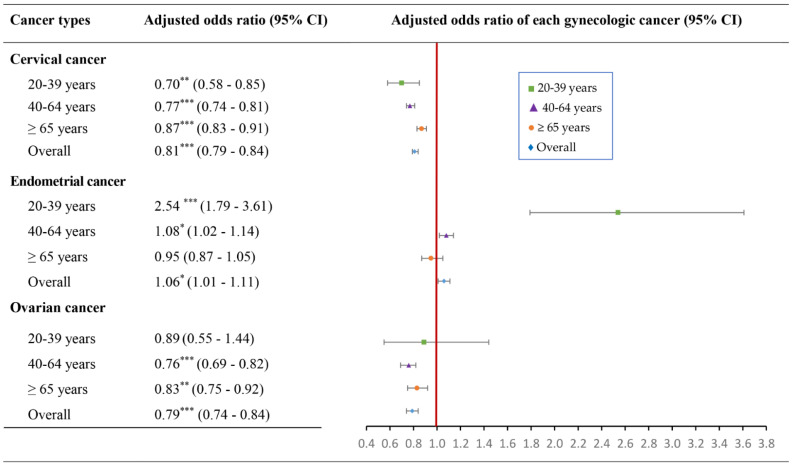
The association of RAAS inhibitors use with cervical, endometrial, and ovarian cancer risks by age groups with adjusted odds ratio. Footnote: * *p* < 0.05, ** *p* < 0.001, *** *p* < 0.0001.

A significantly decreased risk of cervical cancer was found in RASS users in the groups aged 20–39 years, 40–64 years, ≥65 years, and overall, with an aOR of 0.70, 0.77, 0.87, and 0.81, respectively (Figure 3). Meanwhile, RAAS inhibitors were more likely to develop endometrial cancer in the users aged 20–39 years, 40–64 years, and overall, with an aOR of 2.54, 1.08, and 1.06, respectively. The risk of ovarian cancer was significantly decreased in RAAS drug users in the groups aged 40–64 years, ≥65 years, and overall, with an aOR of 0.76, 0.83, and 0.79, respectively.

**Figure 4:**
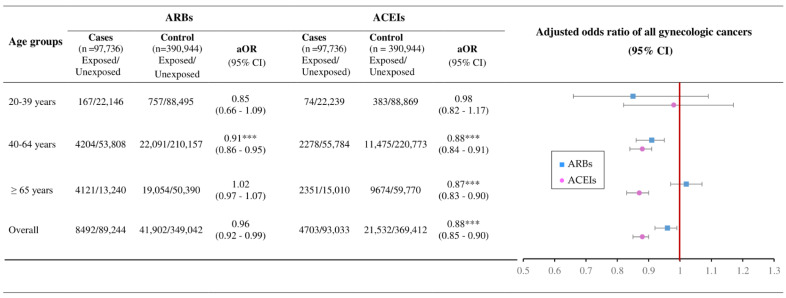
The associations of ARBs and ACEIs use with overall gynecologic cancer risk by age groups with adjusted odds ratios. Footnote: *** *p* < 0.0001.

Figure 4 presents gynecologic cancer risk among ARBs and ACEIs users by age groups. There was a significantly lowered risk of gynecologic cancers in ACEIs users aged 40–64 years, ≥65 years, and overall, with an aOR of 0.88, 0.87, and 0.88, respectively. In addition, ARBs use demonstrated a decreased risk of gynecologic cancers in those 40–64 years, with an aOR of 0.91.

## 3. Discussion

### 3.1. *Main Findings*

This large population-based case-control study highlighted that RAAS inhibitors intake was significantly associated with a decrease in overall gynecologic cancer risks. When stratified by age groups, gynecologic cancer risks were observed to have significant associations with groups aged 40–64 years and ≥65 years. RAAS inhibitors were associated with a lowered cervical cancer risk in 20–64-year-old and ≥65-year-old users, and a reduced ovarian cancer risk in those aged 40–64 years, ≥65 years, overall age group. In contrast, endometrial cancer was shown to be increased risk in users aged 20–64 years, and overall. When stratified by drug groups, ACEIs users were found to have a preventive effect against gynecologic cancers in the groups aged 40–64 years, ≥65 years, and overall age group, whereas ARBs demonstrated a decreased risk of gynecologic cancers in 40–64-year-old users.

### 3.2. *Biological Plausibility*

#### 3.2.1. Postulated Mechanisms of RAAS Inhibitors against Gynecologic Cancers

Mechanisms have been proposed to elucidate the RAAS’s antineoplastic effects against gynecological cancers. First, RAAS inhibitors encourage the potential invasion and release vascular endothelial growth factor (VEGF), which is a potent angiogenic agent in many different types of malignancies [1]. The increase in VEGF production was found in cervical cancer in Siha cell line [1,22,23], endometrial cancer with HEC-1A cell line, [1], and ovarian cancer with SKOV3 cell lines [24]. Second, RAAS affects processes such as proliferation, apoptosis, autography, migration, inflammation, oxidative stress, or angiogenesis [25]. In cervical, ovarian [26], and endometrial carcinomas, altered expression of the system’s peptides and receptors was seen [27,28]. This mechanism was demonstrated in in vitro studies [27,28,29,30,31]. Third, mRNA of RAAS receptors were highly expressed in endometrioid carcinomas and their adjacent endometrium, suggesting that these receptors may play a role in development of endometrial cancer [19]. Some previous studies indicated that body mass index (BMI) and are most significantly linked to endometrial cancer incidence and mortality [32,33,34]. The association between obesity and endometrial cancer can be explained by mechanistic pathways. Visceral fat is a complex endocrine organ that contains adipocytes and preadipocytes as well as stromal, neuron, stem, and macrophage infiltration. Together, they release a variety of adipokines that have both localized and systemic effects, promoting carcinogenesis and enhancing endometrial proliferation [35,36,37]. In addition, adipose tissue is also a source of mesenchymal stem cells, which can be used to promote the development and growth of tumors [38,39]. Four, the overexpression of mRNA and KDR (kinase domain-containing receptor) protein itself has been proposed for the mechanism related to RAAS and gynecological cancer risk. The concentration of mRNA and KDR has been shown in ovarian cancer [40,41,42,43].

In this study, our findings indicated a lowered overall risk of gynecologic cancers in RAAS inhibitors users. Lee SH et al. (2022) conducted a population-based cohort study in Korea and indicated that RAAS inhibitors use was not associated with gynecologic cancers [44]. A meta-analysis of observational studies found no preventive effect of RAAS against gynecologic cancers [45]. Inconsistences between our finding and other studies may be due to the differences in study design, and adjusted confounders.

#### 3.2.2. Postulated Mechanisms of ARBs/ACEIs against Gynecologic Cancers

When stratified by drug groups, ACEIs use was found to have a preventive effect against gynecologic cancers in the groups aged 40–64 years, ≥65 years, and overall age group, whereas ARBs demonstrated a decreased risk of gynecologic cancers in 40–64 year-old users. These results can be supported by some possible mechanisms. In general, ARBs and ACEIs, being potent angiogenic agents in several types of malignancies [1], often encourage invasive potential and VEGF production, which in turn boost angiogenesis and pro-tumorigenic transcription factors [22,23]. These medications also promote inflammation and participate in metastasis, invasion, and migration processes [13,31]. While in vitro and in vivo studies presented that up-regulation of ACEIs was beneficial for establishing local tumor angiogenesis, ARBs may be able to affect angiogenic pathways via restraining cancer cell proliferation and enhancing medication delivery [46,47].

A previous study reported that losartan (ARB) played a vital role in enhancing drug delivery and efficacy via decreasing solid stress, tumor hypoxia, extracellular matrix and augmenting vascular perfusion [48]. This finding contributed to clarifying the physiological mechanism in our study. Another study showed that increasing the ACEI activity remained unexplicit, it might be linked to aging [49]. However, some researchers had suggested that the level of ACEI serum could be used to detect disseminated germinoma tumors and track the effectiveness of treatment [50].

A retrospective cohort study conducted by Cho MA et al. (2020) among Korean patients with ovarian cancer revealed that those who used ARBs were associated with 35% decreased risk of disease progression and recurrence in ovarian cancer [13]. Likewise, women taking ACEIs was found to be associated with the lowest risk of gynecologic tract cancer [15]. A network meta-analyses and trial sequential analyses of 324,168 participants from randomized trials, nevertheless, showed ACEIs/ARBs use were not associated with risk of all cancers [51]. In addition, a population-based cohort study in Denmark demonstrated that no risk reductions were observed for ACEIs and female reproductive tract [52]. These differences could be because of the study population, sample size, and adjusted confounders. Further investigations are encouraged to clarify the significance of ARBs and ACEIs use and gynecological cancers by stratification of age.

This present study has several strengths: First, patients’ information was gathered from a reliable registry that included diagnoses, prescriptions, and definitions of cancer. Secondly, the database contained a large population, therefore, we were able to categorize individuals into age groups. Finally, we considered potential confounding variables that may be associated with gynecologic cancer risks.

However, our study has several limitations. First, this study found associations between RAAS inhibitors and gynecologic cancer risks rather than causality. The findings gave prospective medication-cancer signals that clinicians or researchers can utilize to identify the mechanisms or their causality in the future. Second, information such as patient lifestyles, medication adherence, laboratory data, etc., were not accessible for our analysis. Third, this study could not include some risk factors, including hormone replacement treatment, oral contraception, HPV infection or immunization, hypertension, hyperinsulinemia, number of pregnancies/infertility, BMI, obesity etc.

## 4. Materials and Methods

### 4.1. Data Sources

Data were provided by Health and Welfare Data Science Center (HWDC), which is established by Taiwan’s Ministry of Health and Welfare (MOHW). HWDC contain de-identified claims data of the National Health Insurance (NHI) beneficiaries [53], which covers 99.9% of the Taiwanese population [54]. Now, it provides more than 100 different databases for research, such as Ambulatory Care Expenditures by Visits, Inpatient Expenditures by Admissions, Details of Ambulatory Care Orders, Details of Inpatient Orders, Cause of Death Data, Taiwan Cancer Registry, and so on. In this study, medication and diagnosis data (2000–2016) were retrieved from HWDC, and cancer is confirmed by Taiwan Cancer Registry (TCR) (1979–2016) (Figure 1). The cancer diagnoses in this study were identified from validated International Classification of Diseases for Oncology, 3rd Edition (ICD-O-3) codes and linked to the pathological data. The study was approved by the Joint Institutional Review Board of Taipei Medical University (TMU-JIRB), Taipei, Taiwan (approval number: N202003609).

### 4.2. Definition of Case and Control

This study includes all newly diagnosed female patients with gynecologic cancers from 1 January 2002 to 31 December 2016. Gynecologic cancers were defined based on the International Classification of Diseases, 9th revision, Clinical Modification (ICD-9-CM) (e.g., ICD-9-CM codes 180 for cervical cancer, 182 for endometrial cancer, and 183 for ovarian cancer). The initial date of diagnosis with gynecologic cancers was determined as the index date. Controls were defined as those without any cancer diagnosis between 2000 and 2016. Each eligible case would match with four controls using the propensity score from age, sex, and year of diagnosis. Controls assigned the same index date with their matched cases [55]. We excluded patients under 20 years or with inconsistent data.

### 4.3. RAAS Users

Medications were extracted from the details of ambulatory care orders in the HWDC database. Medication information, including NHI drug codes, drug names, drug dosage, frequency, dispensing date, the total daily dose, and so on. ARBs (C09A), and ACEIs (C09C) were classified using Anatomical Therapeutic Chemical (ATC) codes (see Appendix A). The analyses of ARBs, and ACEIs exposure were conducted only before the cancer diagnosis (e.g., index date). We took into account the patients’ prior exposure to ARBs and ACEIs or not. Therefore, individuals who had received prescriptions for ARBs and ACEIs for at least 60 days within the two years before the index date were categorized as ARB and ACEI users. We defined non-users who had never been prescribed any RAAS drug (ARBs or ACEIs) or prescribed less than 60 days.

### 4.4. Confounding Factors

Comorbid conditions, Charlson Comorbidity Index, and other drugs, such as metformin (ATC: A10BA02) [56,57,58], aspirin (ATC: B01AC06) [58,59,60], and statin (ATC: C10AA) [61] were regarded as potential confounders in our analysis (Table 1). Patients who had been prescribed aspirin, metformin, and statin for at least two months (e.g., 60 days) in the two years before to the index date were considered to have been exposed to those medications.

### 4.5. Statistical Analysis

We applied the McNamara test and paired *t*-test to test the difference between the case and control groups [62]. Conditional logistic regression with 95% confidence intervals (CIs) was utilized to identify the associations of RAAS inhibitors, ARBs, and ACEIs use with gynecologic cancer risks [63]. The models were categorized into different age groups, such as aged ≥20 years, 20–39 years, 40–64 years, and ≥65 years. We utilized SAS v.9.4 software (SAS Institute Inc., Cary, NC, USA) for statistical analysis. A *p*-value ≤ 0.05 was regarded as statistically significant.

## 5. Conclusions

Our finding highlighted that RAAS inhibitors use was significantly associated with decreased risks in overall gynecologic cancers. When RAAS inhibitors users were stratified by age, gynecologic cancer risks were associated with groups aged 40–64 years and ≥65 years. Users of RAAS inhibitors were shown to have a significantly lower risk of cervical cancer in the 20–64 and ≥65-year-old age groups, and a lower risk of ovarian cancer in the 40–64, ≥65-year-old age groups, and overall age group. However, endometrial cancer was observed to be increased risk in the groups aged 20–39 years, 40-64 years, and overall. The significantly reduced risks of gynecologic cancers were associated with ACEIs users in the groups aged 40–64 years, ≥65 years, and overall, and ARBs users aged 40–64 years. Further clinical research are encouraged to establish the causality and confirm mechanism of the associations identified in this study.

## Figures and Tables

**Table 1:** Demographic characteristics of gynecologic cancer cases.

Characteristics	Cases (With Cancer) (*n* = 97,736)	Controls (Without Cancer) (*n* = 390,944)
Age		
Mean ± SD	50.81 ± 13.86	50.81 ± 13.86
20–39 y, *n* (%)	22,313 (22.83)	89,252 (22.83)
40–64 y, *n* (%)	58,062 (59.41)	232,248 (59.41)
>=65 y, *n* (%)	17,361 (17.76)	69,444 (17.76)
Comorbid conditions, *n* (%)		
Myocardial infarction	194 (0.20)	774 (0.20)
Congestive heart failure	1473 (1.51)	6331 (1.62)
Peripheral vascular disease	602 (0.62)	2932 (0.75)
Cerebrovascular disease	3723 (3.81)	17,466 (4.47)
Dementia	627 (0.64)	3427 (0.88)
Chronic pulmonary disease	2680 (2.74)	12,665 (3.24)
Rheumatic disease	1363 (1.39)	6472 (1.66)
Peptic ulcer disease	10,245 (10.48)	49,475 (12.66)
Liver disease	5058 (5.18)	24,268 (6.20)
Diabetes	11,622 (11.89)	55,471 (14.19)
Hemiplegia or paraplegia	157 (0.16)	770 (0.20)
Renal disease	2019 (2.07)	8880 (2.27)
CCI score		
Mean *±* SD	0.46 *±* 0.82	0.52 *±* 0.92
Other drugs, *n* (%)		
Metformin	6817 (6.97)	33,423 (8.55)
Aspirin	6226 (6.37)	29,946 (7.66)
Statin	7613 (7.79)	40,040 (10.24)

CCI, Charlson comorbidity index.

## Data Availability

Restrictions apply to the availability of these data. Data were retrieved from databases of Health and Welfare Data Science Center and are accessible with the approval of of Taiwan’s Ministry of Health and Welfare.

## Abbreviations

aOR	Adjusted odds ratio
ACEIs	Angiotensin-converting enzyme inhibitors
ARBS	Angiotensin receptor blockers
ATC Classification	Anatomical Therapeutic Chemical classification
CCI	Charlson comorbidity index
CI	Confidence interval
HWDC	Health and Welfare Data Science Center
ICD-9-CM	International Classification of Diseases, 9th revision, Clinical Modification
KDR	Kinase domain-containing receptor
MOHW	Ministry of Health and Welfare
NHI	National Health Insurance
TCR	Taiwan Cancer Registry
TMU-JIRB	Joint Institutional Review Board of Taipei Medical University
RAAS	Renin-angiotensin-aldosterone system
VEGF	Vascular endothelial growth factor

## Appendix A

**Table A1:** The RAAS inhibitors classification using Anatomical Therapeutic Chemical (ATC) code.

ATC Code	Name	Covered by National Health Insurance in Taiwan
C09AA01	captopril	1995~
C09AA02	enalapril	1995~
C09AA03	lisinopril	1995~
C09AA04	perindopril	1995~
C09AA05	ramipril	1995~
C09AA06	quinapril	1995~
C09AA07	benazepril	1995~
C09AA08	cilazapril	1995~
C09AA09	fosinopril	1995~
C09AA10	trandolapril	Not Available
C09AA11	spirapril	Not Available
C09AA12	delapril	Not Available
C09AA13	moexipril	Not Available
C09AA14	temocapril	Not Available
C09AA15	zofenopril	Not Available
C09AA16	imidapril	2001~
C09CA01	losartan	1998~
C09CA02	eprosartan	2007~
C09CA03	valsartan	1998~
C09CA04	irbesartan	2000~
C09CA05	tasosartan	Not Available
C09CA06	candesartan	2001~
C09CA07	telmisartan	2001~
C09CA08	olmesartan	2004~
C09CA09	azilsartan	2014~
C09CA10	fimasartan	Not Available
